# Botulinum toxin type A inhibits the formation of hypertrophic scar through the JAK2/STAT3 pathway

**DOI:** 10.17305/bb.2024.10906

**Published:** 2024-08-10

**Authors:** Yan Fan, Xuesong Guo, Yu Tian, Jie Li, Hongwei Xi

**Affiliations:** 1 Department of Paediatrics, Shanxi Medical University, Shanxi, China; 2 Department of Burns and Plastic Surgery, Children’s Hospital of Shanxi (Women Health Center of Shanxi), Shanxi, China; 3 Department of Orthopedics, Children’s Hospital of Shanxi (Women Health Center of Shanxi), Shanxi, China; 4 Department of General Surgery, Children’s Hospital Affiliated to Shanxi Medical University, Shanxi, China

**Keywords:** Botulinum toxin type A (BTA), JAK2/STAT3 pathway, fibrosis, hypertrophic scar (HS)

## Abstract

Hypertrophic scar (HS) is a fibrous proliferative disorder that occurs in the dermis after skin injury. Studies have confirmed that Botulinum toxin type A (BTA) is effective in scar prevention and treatment. However, the specific mechanism remains uncertain. HS fibroblasts (HSFs) and normal skin fibroblasts (NSFs) from the skin tissues of HS patients were isolated and cultured. Western blot analysis was conducted to measure the expression of JAK2/STAT3 pathway-related proteins. HSFs were treated with the JAK2 inhibitor (AG490) or agonist (C-A1). The cell counting kit-8 (CCK-8) assay, EdU staining, scratch-wound assay, and Transwell assay were used to examine the biological properties of HSFs. Western blot, immunofluorescence, and Sirius red staining were used to assess the fibrosis of HSFs. Additionally, a mouse full-thickness wound model was constructed to investigate the role of BTA in wound healing. The results showed that the JAK2 and STAT3 phosphorylation levels were markedly increased in HS tissues and HSFs. AG490 treatment reduced cell viability, proliferation, and migration capacity, and inhibited the fibrosis of HSFs, whereas C-A1 treatment had the opposite effect. BTA treatment inhibited the JAK2/STAT3 pathway. BTA reduced cell viability, proliferation, and migration ability, and inhibited the fibrosis of HSFs, while C-A1 intervention weakened the impact of BTA. Meanwhile, BTA promoted wound healing and reduced collagen deposition in vivo. In conclusion, BTA inhibited the JAK2/STAT3 pathway, which in turn hindered the proliferation, migration, and fibrosis of HSFs, and promoted wound healing in mice.

## Introduction

Hypertrophic scar (HS) is a condition characterized by the abnormal growth of fibroblasts and excessive accumulation of extracellular matrix following a skin injury [1, 2]. Statistically, the incidence of HS due to burns or trauma to the skin ranges from 40% to 70%, and in burn wounds that do not heal within 21 days, the incidence is 70% or higher [3]. Clinically, HS manifests as local hardening and thickening of the scar, irregular shape, red coloration, and protrusion above normal skin, and is often accompanied by itching, pain, and other discomforts, which severely impacts the physical and mental health of patients [4, 5]. Consequently, reducing or inhibiting scar formation is a major challenge that needs to be addressed.

In recent years, significant progress has been made in the management of HS due to advancements in basic and clinical research. Current clinical treatments for HS include surgical intervention, local injection therapy, photoelectric therapy, and topical drug therapy [6, 7]. However, these treatment options have limitations and have not yet achieved completely satisfactory outcomes.

Botulinum toxin type A (BTA), one of the eight neurotoxins produced by Clostridium botulinum, inhibits the release of acetylcholine, leading to muscle relaxation and paralysis, and consequently reducing wound tension [8, 9]. BTA is widely used to reduce wrinkles and improve facial contours, and it has also been shown to be effective in treating migraines, hyperhidrosis, blepharospasm, and facial asymmetry [10–13]. Moreover, several studies suggest that BTA can effectively prevent and treat scars. Chen et al. [14] found that BTA injections after surgery reduced surgical scarring and improved wound appearance. Zhang et al. [15] demonstrated that BTA injection reduced scar thickness and inhibited collagen expression in a rabbit ear model. However, the specific mechanism by which BTA inhibits HS formation remains unclear.

The Janus kinase (JAK)/signal transducer and activator of transcription (STAT) pathway plays a crucial role in transducing information and is responsible for regulating various biological processes, such as cell growth, division, migration, cytokine production, and inflammation [16–18]. Recent research has noted that the JAK2 inhibitor AG490 hindered the JAK2/STAT3 pathway, which in turn inhibited the abnormal proliferation and fibrosis of keloid fibroblasts, suggesting a potential role for this pathway in the development of HS [19]. Therefore, it is necessary to explore whether BTA exerts its effects by regulating the JAK2/STAT3 pathway.

In this research, we extracted HS fibroblasts (HSFs) and normal skin fibroblasts (NSFs) from the skin samples of HS patients to analyze the expression of JAK2/STAT3 pathway proteins in different tissues and cells. Subsequently, we explored the effects of activating or inhibiting the JAK2/STAT3 pathway on the biological properties and fibrosis of HSFs. Finally, we investigated whether BTA inhibits HS formation by modulating the JAK2/STAT3 pathway. The aim of this research is to elucidate the specific mechanism by which BTA inhibits HS formation and to provide a new reference for the treatment of HS.

## Materials and methods

### Clinical tissue samples

Samples of tissues from eight HS patients and their corresponding healthy skin tissues were collected from the Children’s Hospital of Shanxi. The inclusion criteria for the specimens were: (1) a diagnosis of hyperplastic scarring in the proliferative phase, (2) a disease duration of three months to one year, (3) a scar appearance consistent with typical hyperplastic scar manifestations, (4) no prior treatment before surgery, and (5) no significant restrictions on gender, age, or the distribution of scar lesion sites. The exclusion criteria included: the exclusion of patients with pituitary diseases, adrenal diseases, infectious diseases, immunological diseases, or underlying conditions such as hypertension and diabetes mellitus, as well as the exclusion of infections and ulcerated wounds. Approval for this research was obtained from the Ethics Committee of the Children’s Hospital of Shanxi, and written informed consent was provided by all sample providers. The skin tissue samples were collected and placed in a container with sterile PBS at a low temperature, then quickly transferred to the laboratory for processing. The collected HS and NS tissue specimens were divided into two portions. One portion was used to isolate and culture HSFs and NSFs. The other portion of skin tissue was cleared of blood and subcutaneous adipose tissue, cut into small pieces, and transferred into a freezing tube for tissue protein extraction as quickly as possible.

### Isolation and culture of HSFs and NSFs

The HS and NS tissue blocks were washed twice with sterile PBS containing 1% penicillin–streptomycin double antibody (Gibco, Grand Island, NY, USA) under an ultraclean bench, retaining only the dermal tissues and removing the other parts. The dermal tissues were immersed in DMEM medium (Gibco) supplemented with 10% fetal bovine serum (Gibco) and then trimmed into tissue blocks approximately 3 mm × 3 mm × 3 mm in size. The tissues were digested overnight at 4 ^∘^C with 0.25% trypsin. The digestion was terminated by adding sterile PBS to dilute the trypsin, and the tissues were rinsed twice with PBS. The tissue blocks were then transferred to Petri dishes containing a complete medium. On day 4, the Petri dishes were examined under an inverted microscope to check for medium contamination and for cells migrating out from the edges of the tissue blocks. The old medium was carefully removed by pipetting, the tissue blocks were washed with sterile PBS containing 1% penicillin–streptomycin dual antibody, and a fresh complete medium was added, just enough to cover the tissue blocks. The medium was replaced every 3–4 days. Passaging was performed at a 1:3 ratio once the cells reached 80% confluence. The cell culture conditions were maintained at 37 ^∘^C with 5% CO_2_

### Cell counting kit-8 (CCK-8) assay

HSF cells were seeded in 96-well culture plates (1.5 × 10^4^ cells/well). Once the cells had attached, the previous medium was replaced with a new one containing the JAK2 inhibitor AG490 (50 µM, HY-12000, MedChemExpress, Monmouth Junction, NJ, USA), the JAK2 agonist Coumermycin A1 (C-A1, 10 µM, HY-N7452, MedChemExpress), or BTA (2, 4, or 8 units/mL, BOTOX^®^, Allergan, Irvine, CA, USA). After 0, 24, 48, or 72 h of incubation, a complete medium (100 µL) containing 10% CCK-8 reagent (C0038, Beyotime, Shanghai, China) was added to each well. Following a 2-h incubation at 37 ^∘^C in a light-protected incubator, the OD450 value was measured using a microplate reader (Thermo Fisher Scientific, Waltham, MA, USA).

### EdU staining

The EdU Cell Proliferation Detection Kit (C0071S, Beyotime) was used to identify the proliferation of HSFs. Following various treatments, the culture medium was removed, rinsed twice with PBS, and subsequently exposed to 10-µM EdU staining solution for 1 h, protected from light. The cells were then rinsed twice with PBS and exposed to 4% paraformaldehyde (Solarbio, Beijing, China) for 20 min. PBS containing 0.3% Triton X-100 (Sigma-Aldrich, St. Louis, MO, USA) was added to permeabilize the cells for 10 min. A Click reaction solution (Invitrogen, Carlsbad, CA, USA) was incubated in the dark for 30 min, followed by the DAPI staining solution (Invitrogen) for about 10 min. After anti-quenching sealing, the cells were observed and photographed under a fluorescence microscope.

### Scratch-wound assay

A cell suspension (1 mL, 1×10^6^ cells/mL) was aspirated with a sterile pipette and inoculated into 6-well plates that had horizontal lines drawn in advance. After the cells had completely adhered and reached more than 80% confluence, a 20-µL sterile pipette tip was used to gently draw a vertical line perpendicular to the horizontal line on the underside of the cell culture plate. Floating cells were rinsed off twice and resupplied with a serum-free medium containing different treatments. Wound healing was observed at 0 and 24 h after the scratch, and the cell migration rate was subsequently calculated.

### Transwell assay

Cells were routinely digested, collected, and suspended in a serum-free medium containing different treatments. A cell suspension (200 µL, 1×10^5^ cells/mL) was added to the upper chamber, followed by the addition of DMEM medium supplemented with 15% fetal bovine serum in the lower chamber. The cells were incubated for 24 h. The Transwell inserts were removed, rinsed twice with PBS, exposed to 4% paraformaldehyde for 30 min, and then stained with 0.1% aqueous crystal violet (C0121, Beyotime) for 10 min. A cotton swab was used to gently wipe away unmigrated cells from the upper section of the chambers. The fields of view were randomly selected, photographed with an inverted microscope, and the number of migrated cells was counted.

### Immunofluorescence

HSF cells were inoculated in culture dishes at a density of 2×10^4^ cells/mL. After the cells had completely adhered and grown to more than 80% confluence, the culture medium was aspirated, and 4% paraformaldehyde was added to the dishes for 20 min. The cells were permeabilized with 0.3% Triton X-100 (Sigma-Aldrich) for 10 min. Afterward, 5% bovine serum albumin (Sigma-Aldrich) was added to cover the cells for 30 min. The cells were then incubated overnight at 4 ^∘^C with an alpha-smooth muscle actin (α-SMA) primary antibody (ab124964, 1:500, Abcam, Cambridge, MA, USA). On the following day, FITC-labeled secondary goat anti-rabbit IgG (31460, 1:10,000, Invitrogen) was added and incubated for 1 h at 37 ^∘^C, protected from light. After adding the DAPI staining solution and incubating the samples for 10 min, the fluorescence was observed with a fluorescence microscope within 1 h of sealing. The fluorescence intensity was obtained by processing the images with Image J software.

### Sirius red staining

HSF cells were exposed to 4% paraformaldehyde for 30 min and rinsed with PBS. The cells were then stained with 0.1% Sirius red (MM1004, Maokangbio, Shanghai, China) for 1 h and washed with tap water for 1 min. Hematoxylin (C0107, Beyotime) was used to counterstain for 1 min, followed by a 1-min rinse with tap water. After air drying, the cells were examined under a microscope and photographed. Image J software was used to analyze the images for collagen secretion expression.

### The construction of a mouse dorsal whole-layer trauma model

Healthy BALB/c mice were purchased from Vitalriver (Beijing, China) and acclimatized for one week at a constant temperature of 22 ^∘^C. Sodium pentobarbital (50 mg/kg, Sigma-Aldrich) was used to anesthetize the mice via intraperitoneal injection. Once a stable respiratory state was observed, the dorsal hairs were removed and smeared with 8% sodium sulfide, which was washed off with water after 5 min. On the following day, the mice were re-anesthetized, disinfected with povidone–iodine, and a square area with a side length of 1 cm was measured on the lower middle part of the back. The entire skin of the area was removed with scissors, resulting in a full-thickness wound with an area of 1 cm^2^ on the back. Referring to the method of Hu et al. [20], the mice were randomly divided into Control and BTA groups (*n* ═ 5). In the Control group, 200 µL of sterile PBS solution was injected subcutaneously at the periwound level, whereas in the BTA group, 200 µL of BTA (1 unit/g, diluted with PBS) was injected for five consecutive days. Periwound photographs were taken on days 0, 3, 5, 7, 10, and 14, respectively. The mice were euthanized on day 14, and wound tissues were collected for histologic analysis.

### Hematoxylin Eosin (H&E) staining

The wound tissues were exposed to 4% paraformaldehyde, embedded in paraffin, and routinely sectioned (thickness of 4–5 µm) according to the HE Staining Kit (C0105S, Beyotime). Sections were deparaffinized in xylene (Sigma-Aldrich) for 10 min and sequentially placed in 100%, 90%, 80%, and 70% alcohol for 2 min each for gradient dehydration. Hematoxylin staining solution was applied for 5 min, then rinsed in distilled water for 5 s, followed by the addition of a differentiation solution for 5 s and another 10-s rinse. Eosin solution was added to stain for 1 min, followed by gradient dehydration in 70%, 80%, 90%, and 100% alcohol for 10 s each. The sections were soaked in xylene for 5 min for transparency, sealed with neutral gum, and observed under an inverted microscope.

### Masson staining

Wax blocks of mouse wound tissues were routinely sectioned, and after deparaffinization, the sections were stained with a Masson trichrome staining kit (G1340, Solarbio). The sections were first treated with Weigert iron hematoxylin staining solution for 10 min, followed by exposure to Masson bluing solution for 5 min and rinsing with distilled water. The sections were then stained with Ponceau S solution for 10 min, rinsed, and placed in an aniline blue staining solution. After gradient ethanol dehydration and xylene clearing, the sections were sealed with neutral gum after drying and then examined microscopically for wound tissue fibrosis.

### Western blot

RIPA lysate (P0013B, Beyotime) was used to lyse cells or tissues to extract proteins, and the BCA kit (P0012, Beyotime) was used to assess protein concentrations. The sample proteins were transferred to PVDF membranes (Invitrogen) after electrophoresis. After rinsing the membranes, they were incubated overnight at 4 ^∘^C with primary antibodies against p-JAK2 (1:1000), JAK2 (AHO1352, 1:5000, Invitrogen), p-STAT3 (1:1000), STAT3 (MA1-13042, 1:5000, Invitrogen), α-SMA (1:10,000), Collagen I (PA1-26204, 1:1000, Invitrogen), or Collagen III (PA5-27828, 1:1000, Invitrogen). On the following day, after rinsing three times, the membranes were incubated with goat anti-rabbit secondary antibody IgG (1:10,000). After exposure and development, the grayscale value of each protein band was assessed using Image J software, with GAPDH (MA1-16757, 1:1000, Invitrogen) serving as the internal reference.

### Ethical statement

This research was approved by the Children’s Hospital of Shanxi.

### Statistical analysis

All experiments were performed at least three times, and the results were documented as the mean ± standard deviation. SPSS 26.0 software (IBM SPSS Statistics 26) was used for statistical analysis. Student’s *t*-test was used to evaluate differences between the two groups, while ANOVA was applied for comparisons among multiple groups. Prism software (GraphPad 9.0) was used for plotting. *P < 0.05 was considered to indicate a significant difference.

## Results

### JAK2/STAT3 pathway shows activation in HS tissues and HSFs

**Figure 1.**
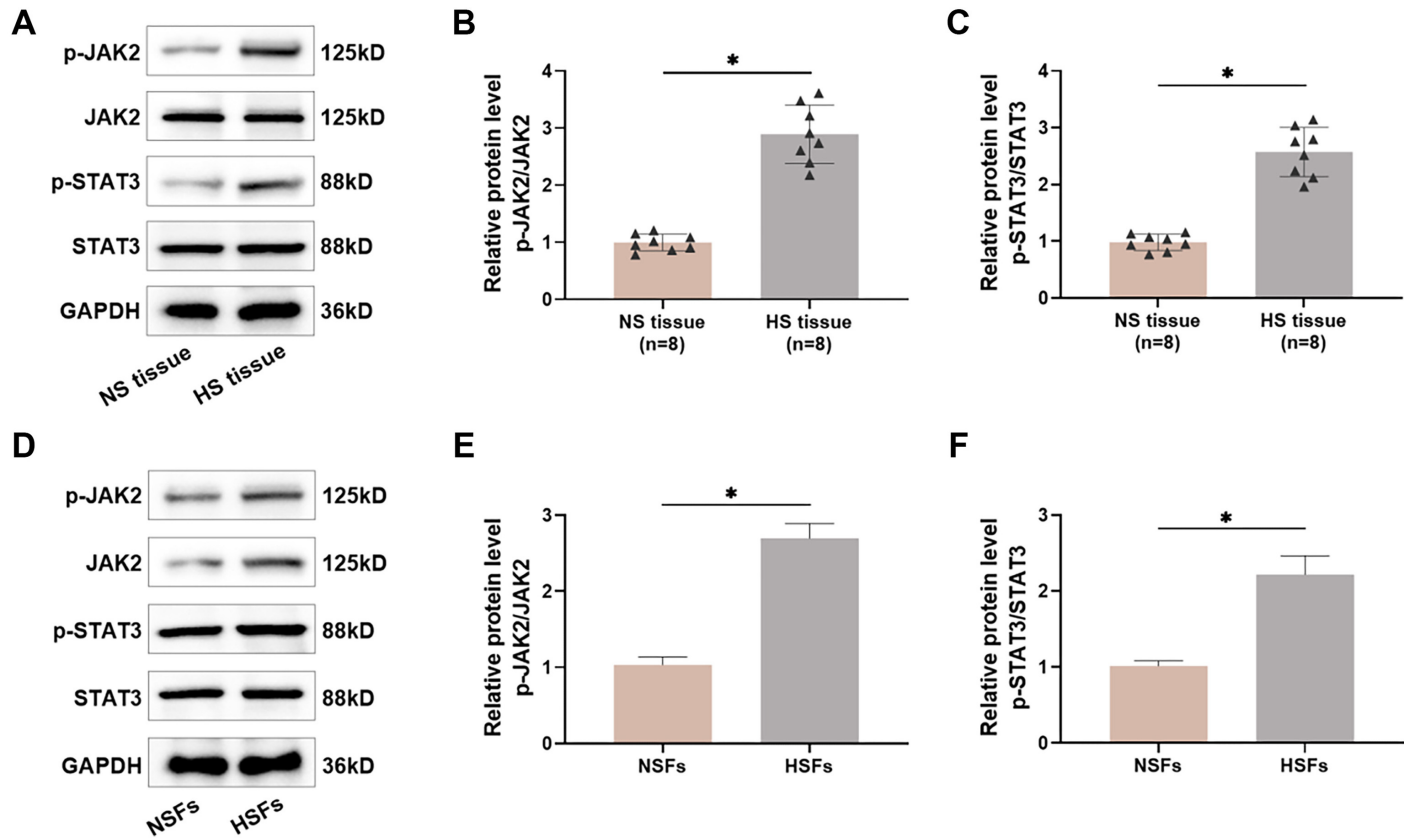
**The JAK2/STAT3 pathway shows activation in HS tissues and HSFs.** (A–C) The levels of JAK2/STAT3 pathway proteins in different tissues were quantified utilizing Western blot, *n* ═ 8; (D–F) The levels of JAK2/STAT3 pathway proteins in NSFs and HSFs were quantified utilizing Western blot, *n* ═ 4. *P < 0.05. HS: Hypertrophic scars; HSFs: Hypertrophic scar fibroblasts; NSFs: Normal skin fibroblasts.

We examined the expression of JAK2/STAT3 pathway proteins in different tissues of HS patients by Western blot. The findings revealed a notable increase in the levels of JAK2 and STAT3 phosphorylation in HS tissues compared to NS tissues (Figure 1A–1C). Additionally, HSFs exhibited a marked increase in the levels of JAK2 and STAT3 phosphorylation compared to NSFs (Figure 1D and 1F). These results indicate that the JAK2/STAT3 pathway is active in both HS tissues and HSFs.

### HSF proliferation and migration are linked to the JAK2/STAT3 pathway

**Figure 2.**
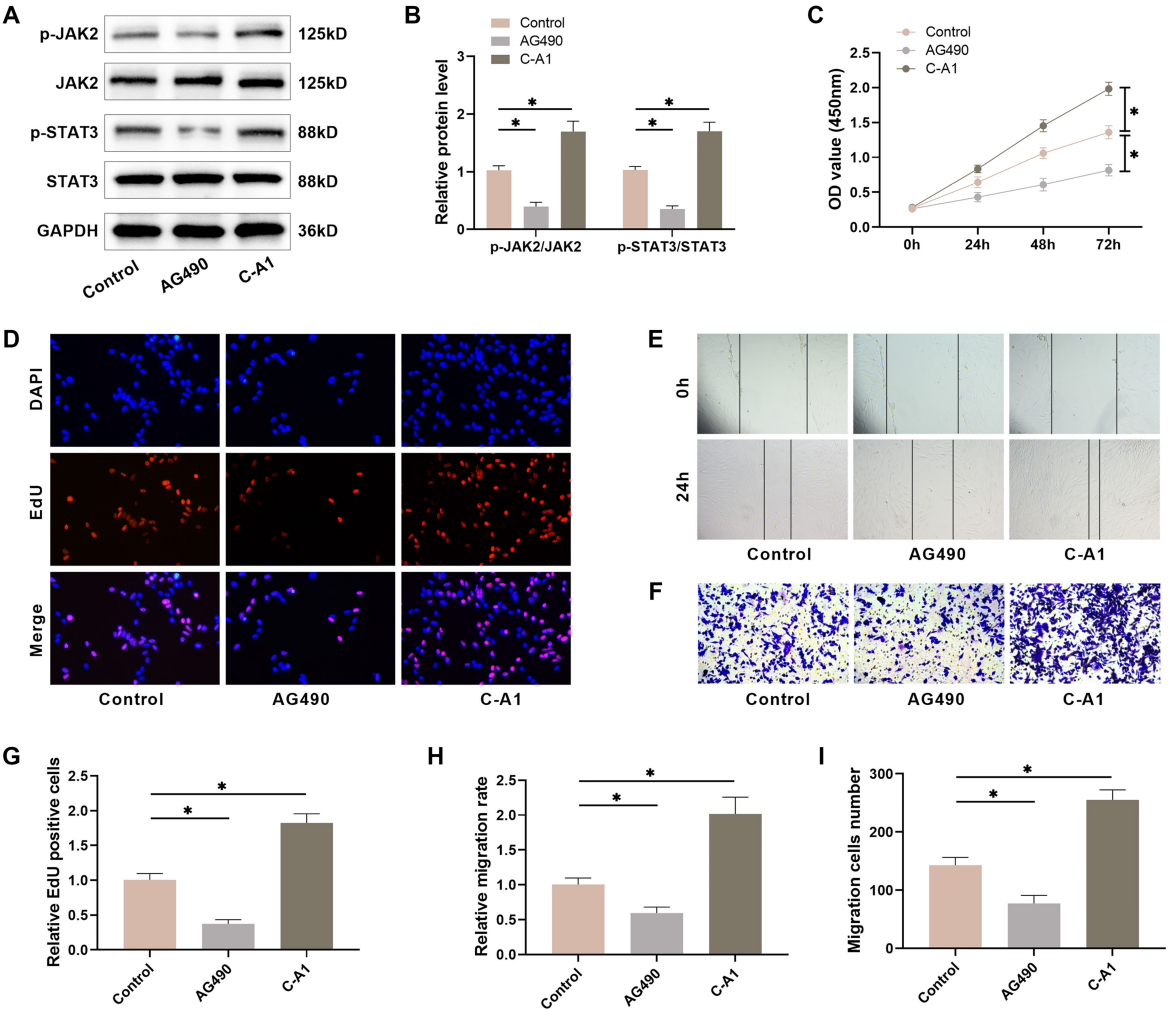
**The JAK2/STAT3 pathway impacts the proliferation and migration of HSFs.** (A and B) The levels of JAK2/STAT3 pathway proteins in HSFs were examined utilizing Western blot; (C) CCK-8 assay was utilized to determine the cell viability; (D and G) EdU staining was utilized to detect HSFs cell proliferation; (E and H) The wound closure rate was scrutinized utilizing scratch-wound assay; (F and I) The amount of migration cells were calculated through Transwell assay. *n* ═ 4, *P < 0.05. HSF: Hypertrophic scar fibroblasts; CCK-8: Cell counting kit-8.

To explore how the JAK2/STAT3 pathway influences HS formation, we treated HSFs with the JAK2 inhibitor AG490 and the agonist C-A1. AG490 treatment notably decreased the levels of JAK2 and STAT3 phosphorylation, while C-A1 treatment markedly increased them (Figure 2A and 2B). Next, we assessed the cell viability of HSFs using the CCK-8 assay to investigate how inhibiting or activating the JAK2/STAT3 pathway affected them. The findings indicated that AG490 treatment notably decreased HSF viability, while C-A1 treatment increased it, with the effect being time-dependent (Figure 2C). EdU staining results showed that AG490 treatment for 48 h hindered HSF proliferation, leading to a notable decline in the number of EdU-positive cells, whereas C-A1 intervention promoted cell proliferation (Figure 2D and 2G). A scratch assay was used to assess the effects of JAK2/STAT3 pathway activation or inhibition on HSF migration. The results indicated that AG490 treatment notably reduced the migratory ability of HSFs, whereas C-A1 treatment facilitated cell migration (Figure 2E and 2H). The Transwell assay results also demonstrated a noticeable decrease in the migratory capacity of HSFs with the suppression of the JAK2/STAT3 pathway, whereas activating this pathway had the opposite effect (Figure 2F and 2I). These results suggest that the JAK2/STAT3 pathway influences the proliferation and migration of HSFs.

### HSF fibrosis is linked to the JAK2/STAT3 pathway

**Figure 3.**
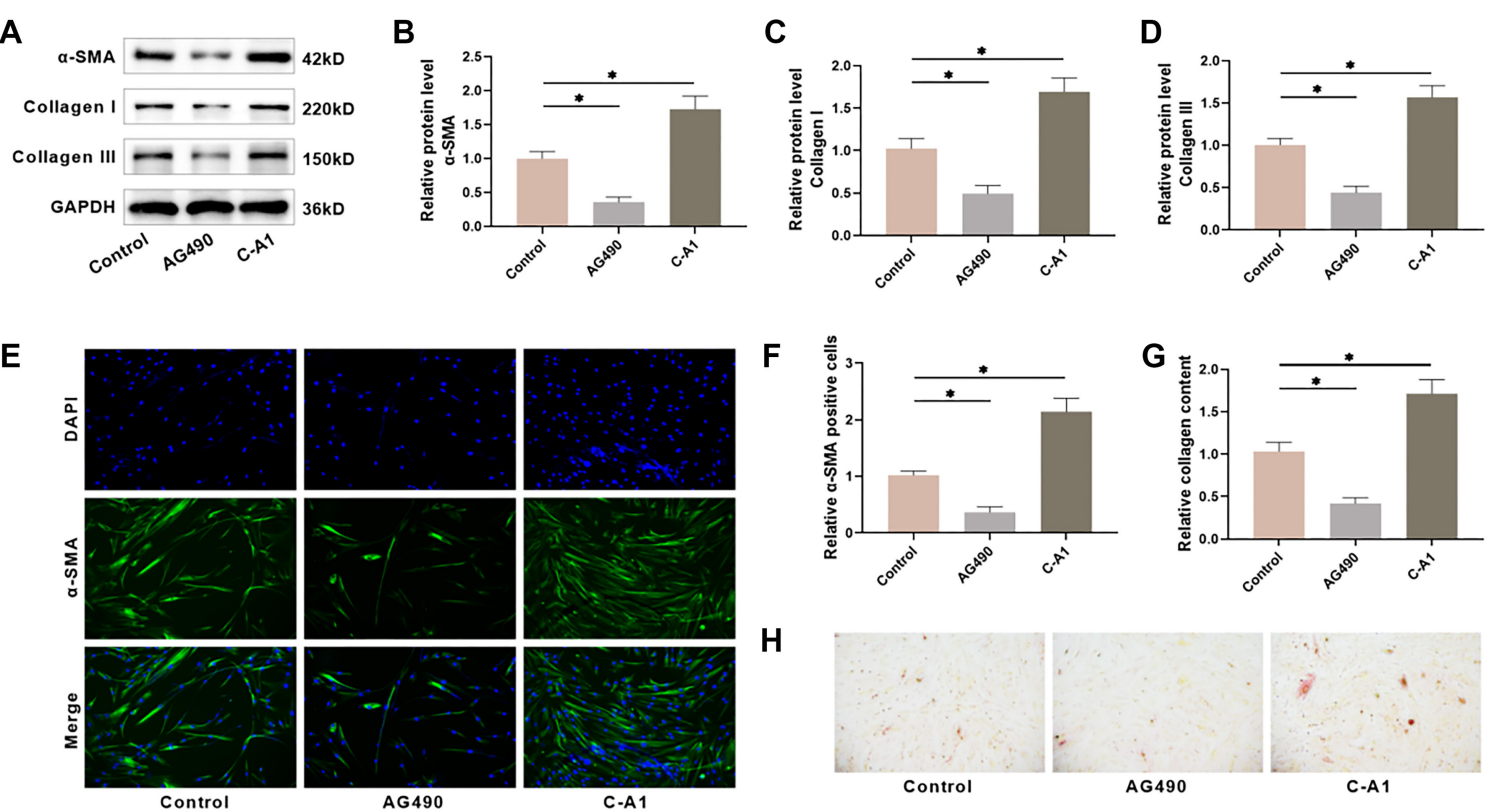
**The JAK2/STAT3 pathway impacts the fibrosis of HSFs.** (A–D) Examining the expression of fibrosis markers in HSFs through Western blot; (E and F) Immunofluorescence was utilized to determine α-SMA levels in HSFs; (G and H) Utilizing Sirius red staining to identify collagen deposition in HSFs. *n* ═ 4, *P < 0.05. HSF: Hypertrophic scar fibroblasts; α-SMA: Alpha-smooth muscle actin.

Next, we investigated how the JAK2/STAT3 pathway influences the fibrosis of HSFs. Western blot analysis revealed a notable reduction in fibrosis markers, such as α-SMA, Collagen I, and Collagen III in HSFs after 48 h of AG490 treatment, whereas C-A1 treatment markedly elevated the levels of these fibrosis markers (Figure 3A–3D). Immunofluorescence experiments showed that AG490 treatment notably reduced the number of α-SMA-positive cells, while C-A1 treatment increased the number of α-SMA-positive cells, in agreement with the Western blot results (Figure 3E and 3F). Additionally, we detected collagen deposition in HSFs by Sirius red staining, which showed that the AG490-treated group had lighter staining, while the C-A1-treated group had darker staining. Quantitative analysis also showed that collagen content in HSFs was notably reduced in the AG490-treated group and significantly increased in the C-A1-treated group (Figure 3G and 3H), indicating the contribution of the JAK2/STAT3 pathway to HSF fibrosis.

### BTA inhibits the JAK2/STAT3 pathway in HSFs

**Figure 4.**
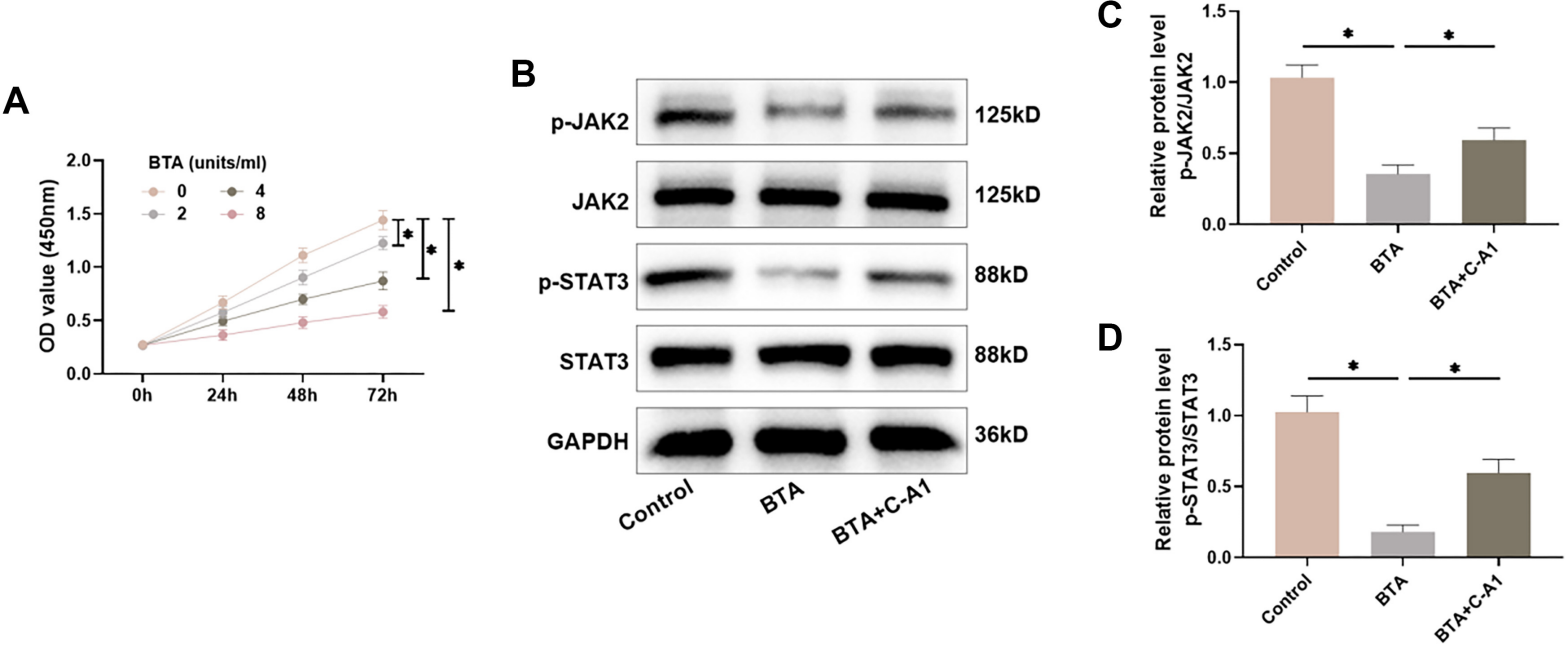
**BTA inhibits the JAK2/STAT3 pathway in HSFs.** (A) CCK-8 assay was utilized to monitor the influence of BTA on cell viability; (B–D) The levels of JAK2/STAT3 pathway proteins in HSFs were examined utilizing Western blot. *n* ═ 4, *P < 0.05. BTA: Botulinum toxin type A; HSF: Hypertrophic scar fibroblasts; CCK-8: Cell counting kit-8.

The effects of different concentrations of BTA (0, 2, 4, and 8 units/mL) on cell viability were examined using the CCK-8 assay. The findings demonstrated that BTA effectively reduced the cell viability of HSFs in a time- and dose-dependent manner (Figure 4A). Therefore, in subsequent experiments, we treated HSFs with 8 units/mL of BTA for 48 h. Notably, the BTA-treated group demonstrated a marked decline in the levels of JAK2 and STAT3 phosphorylation in HSFs, while C-A1 treatment attenuated the effect of BTA (Figure 4B–4D), suggesting that BTA inhibits the JAK2/STAT3 pathway.

### JAK2 agonist reverses the suppressive impact of BTA on HSF proliferation and migration

**Figure 5.**
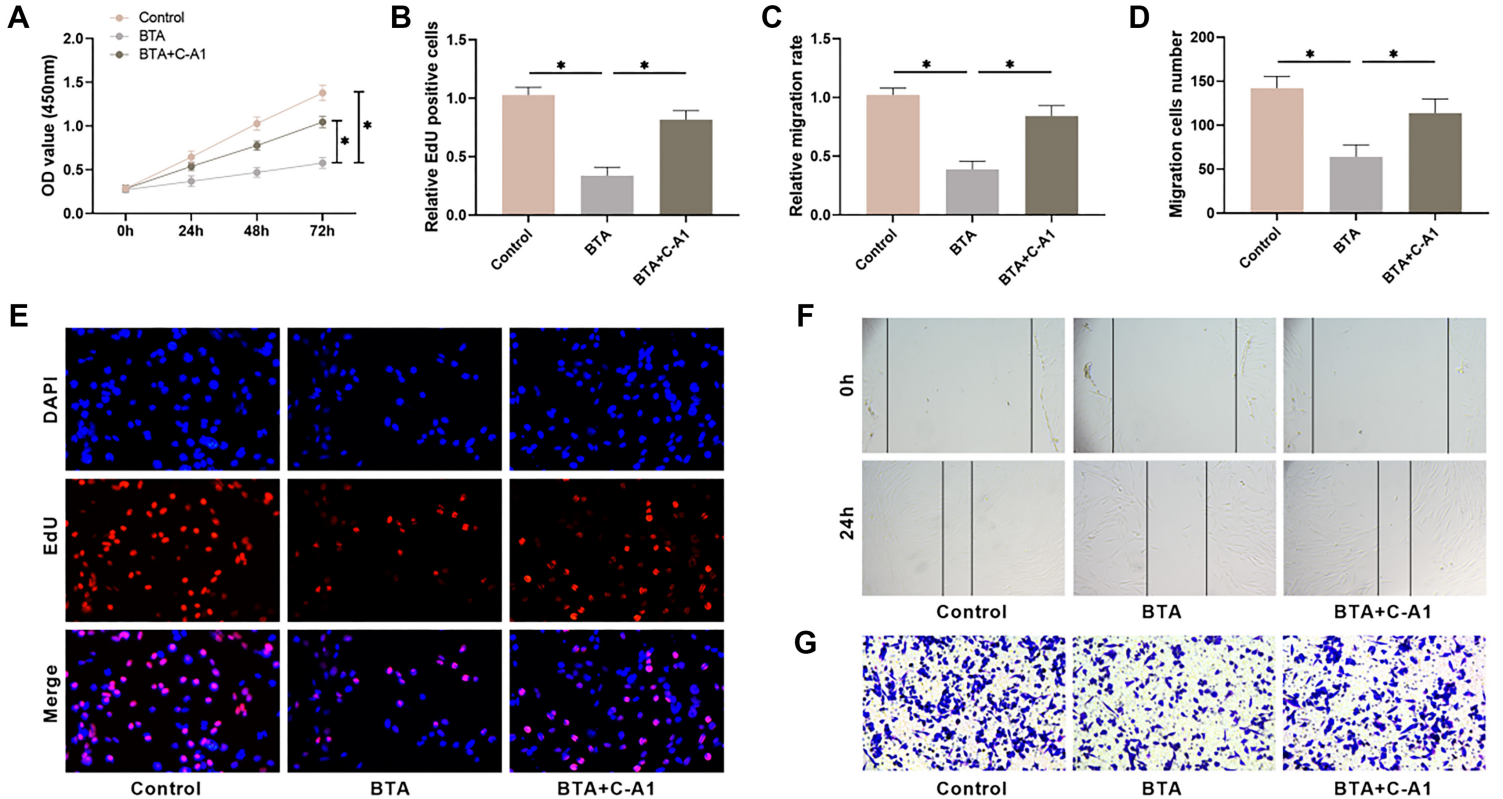
**BTA hinders the proliferation and migration of HSFs and is reversed by JAK2 agonists.** (A) CCK-8 assay was utilized to monitor the cell viability; (B and E) EdU staining was utilized to detect HSFs cell proliferation; (C and F) The wound closure rate was scrutinized utilizing scratch-wound assay; (D and G) The amount of migration cells was calculated through transwell assay. CCK-8: Cell counting kit-8. *n* ═ 4, *P < 0.05. BTA: Botulinum toxin type A; HSF: Hypertrophic scar fibroblasts; CCK-8: Cell counting kit-8.

To determine whether BTA inhibits HS formation by modulating the JAK2/STAT3 pathway, we used C-A1 to intervene in BTA-treated HSFs. CCK-8 assay results revealed a notable decrease in HSF viability after BTA treatment, while C-A1 intervention attenuated the effect of BTA (Figure 5A). EdU staining showed a significant reduction in EdU fluorescence intensity and the number of EdU-positive cells in BTA-treated HSFs, while C-A1 intervention attenuated the effect of BTA (Figure 5B and 5E). Furthermore, we found that BTA treatment decreased the migration rate of HSFs (Figure 5C and 5F), resulting in a marked decrease in the number of migrating cells (Figure 5D and 5G), and C-A1 intervention was able to attenuate the inhibitory effect of BTA on HSF migration. These findings indicate that BTA inhibits the JAK2/STAT3 pathway, leading to the suppression of HSF proliferation and migration.

### JAK2 agonist reverses the suppressive impact of BTA on fibrosis in HSFs

**Figure 6.**
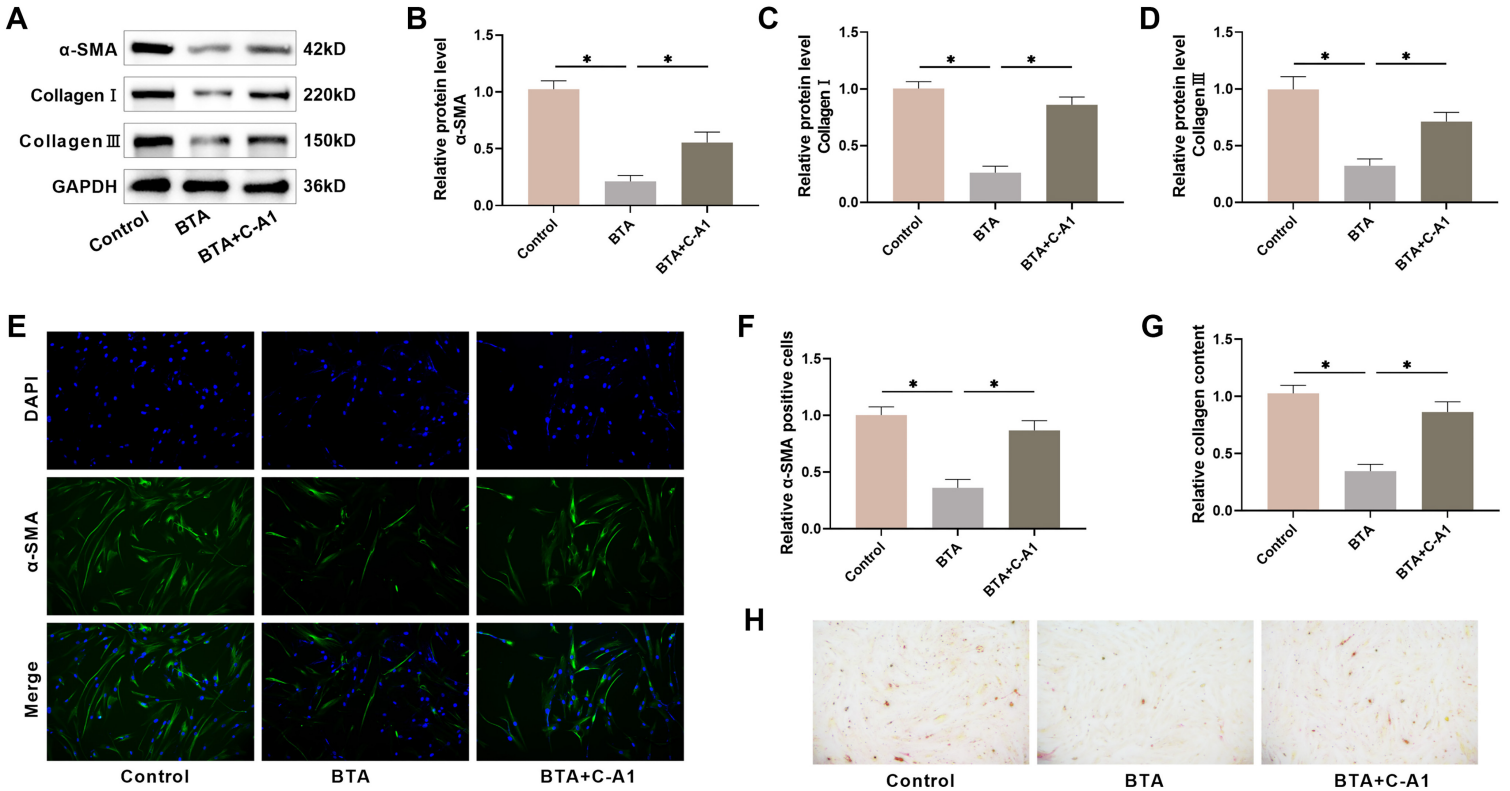
**BTA inhibits fibrosis of HSFs and is reversed by JAK2 agonists.** (A–D) Examining the protein levels of fibrosis markers in HSFs through Western blot; (E and F) Immunofluorescence was utilized to determine α-SMA levels in HSFs; (G and H) Sirius red staining was utilized to identify collagen deposition in HSFs. *n* ═ 4, *P < 0.05. BTA: Botulinum toxin type A; HSF: Hypertrophic scar fibroblasts.

We explored whether BTA inhibits HSF fibrosis by inhibiting the JAK2/STAT3 pathway. Following BTA treatment, Western blot analysis revealed a notable decrease in α-SMA, Collagen I, and Collagen III protein expression in HSFs; however, C-A1 intervention weakened the inhibitory effect of BTA on HSF fibrosis (Figure 6A–6D). Additionally, BTA treatment led to a notable decline in the number of α-SMA-positive cells in HSFs, whereas C-A1 attenuated the effect of BTA, corresponding with the Western blot results (Figure 6E and 6F). Sirius red staining showed that BTA treatment significantly reduced collagen content in HSFs, which was reversed by C-A1 (Figure 6G and 6H). These results further suggest that BTA may prevent HSF fibrosis by inhibiting the JAK2/STAT3 pathway.

### BTA promotes epithelial process to accelerate wound healing by inhibiting the JAK2/STAT3 pathway

**Figure 7.**
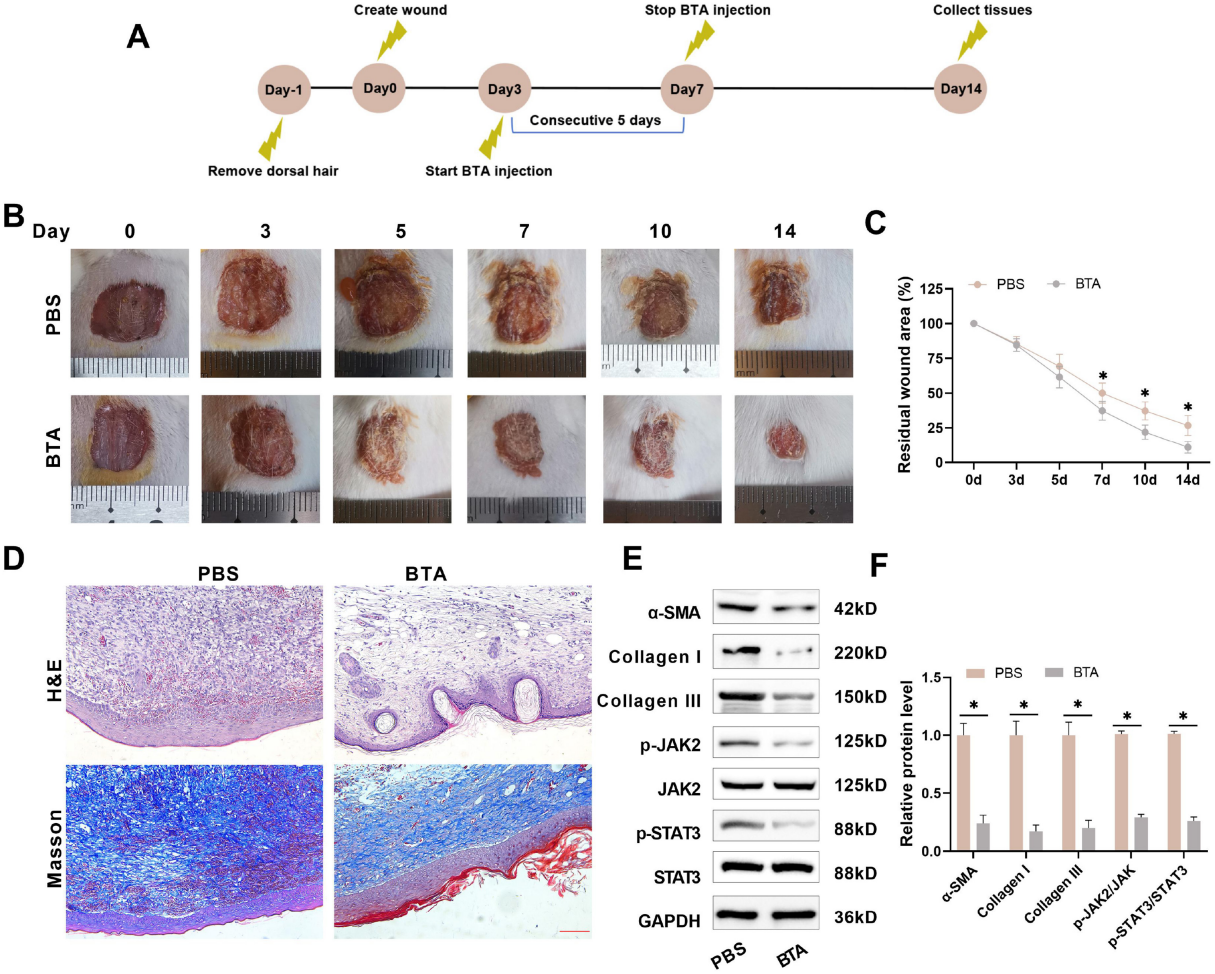
**BTA promotes wound healing and reduces collagen deposition by inhibiting the JAK2/STAT3 pathway.** (A) A mouse whole-layer trauma model was constructed and randomized into Control group (subcutaneous injection of PBS) or BTA group (subcutaneous injection of 1 units/g BTA). Administration was started on day 3 and continued for five consecutive days; (B and C) Photographs of traumatic tissues were taken on days 0, 3, 5, 7, 10, and 14 to document wound area; (D) Mice were executed on the 14th day, and the pathologic structure and collagen deposition of the wounds were observed by H&E and Masson staining (20×, bar=100 µm); (E and F) Western blot examined the levels of α-SMA, Collagen I, Collagen III, and JAK2/STAT3 pathway proteins in traumatic tissues. H&E: Hematoxylin Eosin. *n* ═ 5, *P < 0.05. BTA: Botulinum toxin type A.

Finally, to investigate the role of BTA in wound healing, we constructed a mouse full-thickness wound model following the procedure shown in Figure 7A. As can be seen from the wound images, the wound area in the BTA group was significantly smaller after five consecutive days of administration, indicating that BTA promoted wound healing in the mice (Figure 7B and 7C). H&E and Masson staining showed that BTA treatment promoted the formation of new blood vessels and hair follicles in the wounded tissues and reduced collagen deposition, resulting in a more organized collagen structure (Figure 7D). Western blot results showed that BTA treatment significantly reduced the levels of α-SMA, Collagen I, and Collagen III proteins in the wounded tissue. In addition, the levels of JAK2 and STAT3 phosphorylation were markedly reduced in the wounded tissues after BTA treatment, suggesting that BTA can also inhibit the JAK2/STAT3 pathway in vivo (Figure 7E and 7F). These results further suggest that BTA promotes wound healing and prevents collagen deposition by inhibiting the JAK2/STAT3 pathway in vivo.

## Discussion

In recent years, scholars have conducted many studies on HS, a type of pathological scarring resulting from abnormal tissue repair after skin injury. The condition is primarily caused by enhanced activity and excessive proliferation of fibroblasts [21, 22]. Fibroblasts are the primary cells responsible for wound healing, with HSFs proliferating more vigorously compared to NSFs, and secreting collagen, fibronectin, elastin, and proteoglycans at significantly higher levels [23, 24]. Wound healing progresses through various phases, including inflammation, proliferation, and remodeling [25, 26]. Active cell proliferation and migration characterize the proliferative phase, and the migration of fibroblasts from the wound edges to the center is essential for wound healing [27, 28]. Therefore, inhibiting the proliferation and migration of HSFs is the key to preventing scar proliferation.

The JAK2/STAT3 pathway is a well-known pathway for transmitting cytokine signals within cells and plays a vital role in the inflammatory pathology of several diseases [29, 30]. Sun et al. [31] found that allicin inhibits the JAK2/STAT3 pathway, leading to decreased proliferation, migration, and tube-forming potential of vascular endothelial cells, with the potential to prevent epidural proliferative scarring. Similarly, Singh et al. [32] discovered that the activation of the JAK/STAT3 pathway plays a role in the transdifferentiation of cardiac fibroblasts into myofibroblasts. In our research, the JAK2/STAT3 pathway showed activation in HS tissues and HSFs. Importantly, AG490, a JAK2 inhibitor, reduced the proliferation and migration of HSFs, while C-A1, a JAK2 agonist, had the opposite effect, implying a connection between HS formation and the JAK2/STAT3 pathway, which aligns with the findings of Zhou et al. [19]. Interestingly, BTA treatment markedly decreased JAK2 and STAT3 phosphorylation levels in HSFs and traumatic tissues, suggesting that BTA inhibits the JAK2/STAT3 pathway, which could be an important mechanism by which BTA inhibits HS formation. Moreover, we found that C-A1 intervention attenuated the suppressive influence of BTA on the proliferation and migration of HSFs, confirming that BTA can act by inhibiting the JAK2/STAT3 pathway. Notably, although both BTA and AG490 inhibit the JAK2/STAT3 pathway, BTA also inhibits other pathways, such as TGF-β1/Smad and ERK [33], and PTEN/PI3K/Akt [34], whereas AG490 primarily inhibits the JAK2/STAT3 pathway.

As key effector cells for tissue fibrosis in various organs, myofibroblasts are mainly differentiated from quiescent fibroblasts in human dermal tissues [35, 36]. Myofibroblasts in granulation tissue are activated during proliferative scar formation and promote scar tissue formation, thickening, and stiffness by upregulating α-SMA expression [37]. Additionally, myofibroblasts promote the secretion of collagen, mainly types I and III, leading to excessive deposition of the extracellular matrix, a key factor in HS formation [38, 39]. A previous study indicated that the JAK2 agonist C-A1 activated phosphorylated STAT3, exacerbating oxidative damage and collagen deposition in lung fibroblasts, confirming that the JAK2/STAT3 pathway is associated with fibrosis [40]. Our research revealed that activating the JAK2/STAT3 pathway enhanced α-SMA expression in HSFs, leading to elevated Collagen I and Collagen III secretion, suggesting that the fibrosis of HSFs is linked to the JAK2/STAT3 pathway. The levels of α-SMA, Collagen I, and Collagen III decreased following BTA treatment, suggesting that BTA can inhibit fibrosis in HSFs and traumatic tissues, consistent with observations by Li et al. [33]. Furthermore, Park et al. [41] identified that BTA could inhibit the proliferation, migration, and phosphorylation of HSFs by promoting the phosphorylation of JNK, confirming that BTA may act by activating the JNK pathway. Our study showed that C-A1 intervention attenuated the inhibitory effect of BTA on HSF fibrosis, confirming that BTA inhibits HS formation through the inhibition of the JAK2/STAT3 pathway. In this research, we elucidated the mechanism of BTA in inhibiting HS formation using cellular and mouse full-thickness wound models. However, there are still some limitations. The formation of HS involves multiple signaling pathways, and the crosstalk between the JAK2/STAT3 pathway and other pathways warrants further investigation. BTA is known to inhibit acetylcholine release, but the link between this effect and the JAK2/STAT3 pathway remains unclear. Additionally, it could be later investigated whether BTA affects damaged skin repair by modulating other pathways.

## Conclusion

In summary, the JAK2/STAT3 pathway was activated in HS tissues and HSFs and contributed to the proliferation, migration, and fibrosis of HSFs. BTA suppressed this pathway in HSFs and traumatic tissues, leading to the reduction of proliferation, migration, and fibrosis, as well as the prevention of collagen deposition. This study primarily elucidated the mechanism by which BTA inhibits HS formation and could provide valuable insights for the development of therapies aimed at preventing and treating HS based on BTA.

## Data Availability

The data supporting the findings of this study can be obtained from the corresponding author, upon request.
